# Control of Dog Mediated Human Rabies in Haiti: No Time to Spare

**DOI:** 10.1371/journal.pntd.0003806

**Published:** 2015-06-25

**Authors:** Max F. Millien, Jocelyne B. Pierre-Louis, Ryan Wallace, Eduardo Caldas, Jean M. Rwangabgoba, Jean L. Poncelet, Ottorino Cosivi, Victor J. Del Rio Vilas

**Affiliations:** 1 Ministry of Agriculture, Natural Resources and Rural Development, Port-au-Prince, Haiti; 2 Ministry of Public Health and Population, Port-au-Prince, Haiti; 3 Centers for Disease Control and Prevention, Atlanta, Georgia, United States of America; 4 Servicio de Vigilancia da Saude (SVS), Ministry of Health, Brasilia, Brazil; 5 Pan American Health Organization, Port-au-Prince, Haiti; 6 Pan American Center of Foot-and-Mouth Disease (PANAFTOSA), Pan American Health Organization, Rio de Janeiro, Brazil; The Global Alliance for Rabies Control, UNITED STATES

## Abstract

The American region has pledged to eliminate dog-mediated human rabies by 2015. As part of these efforts, we describe the findings of a desk and field mission review of Haiti’s rabies situation by the end of 2013. While government officials recognize the importance of dog-mediated rabies control, and the national rabies plan adequately contemplates the basic capacities to that effect, regular and sufficient implementation, for example, of dog vaccination, is hampered by limited funding. Compounding insufficient funding and human resources, official surveillance figures do not accurately reflect the risk to the population, as evidenced by the large number of rabid dogs detected by focalized and enhanced surveillance activities conducted by the Ministry of Agriculture, Natural Resources and Rural Development (MARNDR) and the Health and Population Ministry (MSPP) with the technical assistance of the United States Centers for Disease Control and Prevention. Although international support is common, either in the form of on-the-ground technical support or donations of immunobiologicals, it is not comprehensive. In addition, there is limited coordination with MARNDR/MSPP and with other actors at the strategic or operational level due to human resources limitations. Given these findings, the 2015 elimination goal in the region is compromised by the situation in Haiti where control of the disease is not yet in sight despite the best efforts of the resolute national officials. More importantly, dog-mediated rabies is still a threat to the Haitian population.

## Introduction

As part of the regional efforts by the Pan American Health Organization (PAHO) to eliminate dog-mediated rabies in the American region by 2015 [1], a mission led by the Pan American Center of foot-and-mouth disease (PANAFTOSA), a regional center of PAHO with specific responsibilities on the regional control of rabies, visited Haiti in early December 2013. The objectives of the mission were to assess the status of Haiti’s rabies programme as delivered by MARNDR and MSPP, seek opportunities for collaboration between Haiti and Brazil, and explore coordination with the rabies programme conducted by the United States Centers for Disease Control and Prevention (CDC) in Haiti. In what follows, we describe the insights gathered during the mission that comprised a review of the evidence and control plans to date, compare them against the basic capacities of a comprehensive rabies programme, and discuss the findings in the light of the elimination goal in the region by 2015.

## The Context

PAHO’s regional programme of rabies control defines Haiti as a priority country, i.e., countries where canine rabies variants are either circulating or did so in the recent past. The context and capacities of priority countries are diverse, and efforts are undergoing to evaluate the best portfolio of actions to address country-specific limitations [2]. Brazil, for example, remains a priority country because of the persistent, albeit well-circumscribed, occurrence of dog-mediated human rabies in the northeast of the country, in the state of Maranhao [3]. Even with this occurrence, Brazil remains a successful example in the control of dog-mediated rabies, and constitutes an important technical reference in the region. With the aim of promoting south-to-south collaboration, the Ministry of Health of Brazil, the ministry solely responsible for the control of dog-transmitted rabies in the country, assisted PAHO’s mission to Haiti. Specifically on rabies, Brazil has donated human and canine vaccines to Haiti in the past. One of the objectives of the PAHO mission was to explore ways to enhance this collaboration, for example, in the form of direct technical assistance on the ground. Such assistance would complement the activities currently conducted by the CDC. In 2011, in close collaboration with the MARNDR, the CDC initiated a five year rabies infrastructure improvement programme focusing on three critical areas of canine rabies control: surveillance, diagnostics, and education. Collaborations with the MSPP to improve public education on rabies and human rabies surveillance are also ongoing.

With a gross-national-income per-capita of <US$1,035, Haiti is the only country in the American region that is part of the group of low income countries [4]. The other 33 low income countries, as classified by the World Bank, are in Africa and Asia, where dog-transmitted rabies is also endemic and results in thousands of deaths annually [5]. Weak governance and limited resources appear well linked to the persistence of dog rabies [5]. Haiti is also the only country in the region where control of canine rabies is not led by the Ministry of Health (MSPP in Haiti), but by the MARNDR. The MSPP manages health care and rabies prevention in the human population. This separation of responsibilities demands good coordination to ensure, for example, that rabies cases in humans and dogs are communicated between the two Ministries to initiate exhaustive investigations to identify the source of exposure and to implement effective and prompt control measures to prevent further enzootic and cross species transmission. Experience elsewhere shows that this is difficult enough within departments of the same ministry (e.g., health care and epidemiology). A comprehensive evaluation of Haiti’s rabies programme should start by assessing the costs and benefits of this separation of responsibilities between the two ministries.

## Rabies Control Programme: Basic Capacities

An effective rabies control programme requires a number of basic capacities that support interventions at the animal and human fronts. We describe those of Haiti following the structure suggested by [4]. We merely use this scheme to better structure the findings of our mission, and stop short of quantifying the different levels of performance within the capacities as this would have required a much longer mission. Only those capacities that could be discussed during the mission are presented.

### Surveillance

Rabies surveillance and diagnosis in dogs constitutes the main focus of CDC activities in Haiti. Specifically, on diagnosis, the CDC, in collaboration with the NGO Christian Veterinary Mission, has trained, to date, more than 20 MARNDR laboratory personnel in rabies diagnostic methods (Direct Fluorescent Antibody [DFA] and Direct Rapid Immunohistochemistry Test [dRIT]), and improved a diagnostic laboratory in Port-au-Prince with advanced diagnostic equipment. Animal rabies surveillance activities follow a bite-reporting model in which the public and medical providers report bite events to rabies control officers. To support these activities, more than 30 field veterinary and health agents received training in rabies surveillance. Rabies control officers, in turn, attempt to locate the offending animal and conduct a rabies assessment. Animals involved in a bite event are either euthanized and tested or quarantined for 14 days in the owner’s home. Animals reported due to signs of rabies, regardless of human exposure, are tested as well. This passive animal rabies surveillance programme is currently restricted to two of the ten geographical departments of the country (West and Artibonite), chosen primarily for security reasons, and that contribute approximately 50% of the Haitian population. To support activities on the field, the CDC and the NGO Christian Veterinary Mission maintain four Haitian staff in the West Department and three in the Artibonite Department, respectively. The results of these efforts are starting to show the level of under-reporting of canine rabies. Official statistics reported six cases of rabid dogs in 2012 (as of early December 2012) for the country. This compares with 42 rabid dogs identified by the passive surveillance programme in just three communities in Port-au-Prince during the first nine months of the establishment of this surveillance system in 2012.

There is no laboratory-based surveillance of human rabies in Haiti. All diagnoses are based on clinical history. This is due to the current lack of dedicated laboratory facilities for the diagnosis of human rabies, which also impedes the identification of the virus variants and severely limits the evidence available to inform the epidemiology of the disease in the country. Public aversion to autopsy and insufficient numbers of pathologists for collection of samples also limits human rabies surveillance. Alternative, less invasive routes for specimen collection, which may include the trans-foramen magnum or trans-orbital route, as well via skin and hair follicles at the nape of the neck for immunofluorescent staining for viral antigen detection may merit study. To help with this basic capacity, the CDC has conveyed to the MSPP its willingness to assist in human sample collection, shipping of samples, and diagnostic testing at CDC’s rabies laboratory in Atlanta, Georgia, US. To date, only one sample has been shipped to the CDC, which tested negative for rabies.

At a meeting during the mission, MSPP officials reported three human cases in 2013, eight in 2012, 13 in 2011, and one in 2010, all allegedly due to canine rabies. These numbers indicate severe under-reporting when compared with modeling figures that show Haiti as the main contributor of the estimated 200 human deaths by dog-transmitted rabies per year in the region [3]. The above structural limitations do not extend to animal samples that are analysed at the MARNDR rabies laboratory. Despite there being no technical reasons to prevent the use of the MARNDR facilities for human rabies diagnoses, administrative and organizational concerns have prevented the shared utilization of these premises by the two ministries. However, MARNDR’s laboratory is available to receive human samples for diagnosis if and when necessary.

### Dog Vaccination

Dog vaccination remains the most efficient mechanism for the control of dog-mediated rabies as seen throughout the American region where medical authorities have achieved rabies control via mass dog vaccination campaigns [6]. Vaccination failures, e.g., in the form of disruption of annual campaigns or insufficient coverage, have been pointed out as the main cause for reoccurrence of dog rabies and sustained failure to control the disease. In Haiti, dog vaccination has been inconsistently applied. The last mass vaccination campaign was funded and conducted in 2012 by the MARNDR. Approximately 400,000 dogs were vaccinated. For a current human population of approximately 10 million, assuming a relation of one dog per 10 people and aiming to attain 70% of vaccine coverage, 700,000 dogs would have to be vaccinated. The lack of reliable dog population figures (estimates range from 800,000 to 1,200,000 million dogs) makes the assessment of the efficacy of the vaccination campaign difficult, although the persistence of rabid dogs in 2013, as explicitly shown by the CDC figures (above), provides some indication of its limited efficacy.

**Fig 1:**
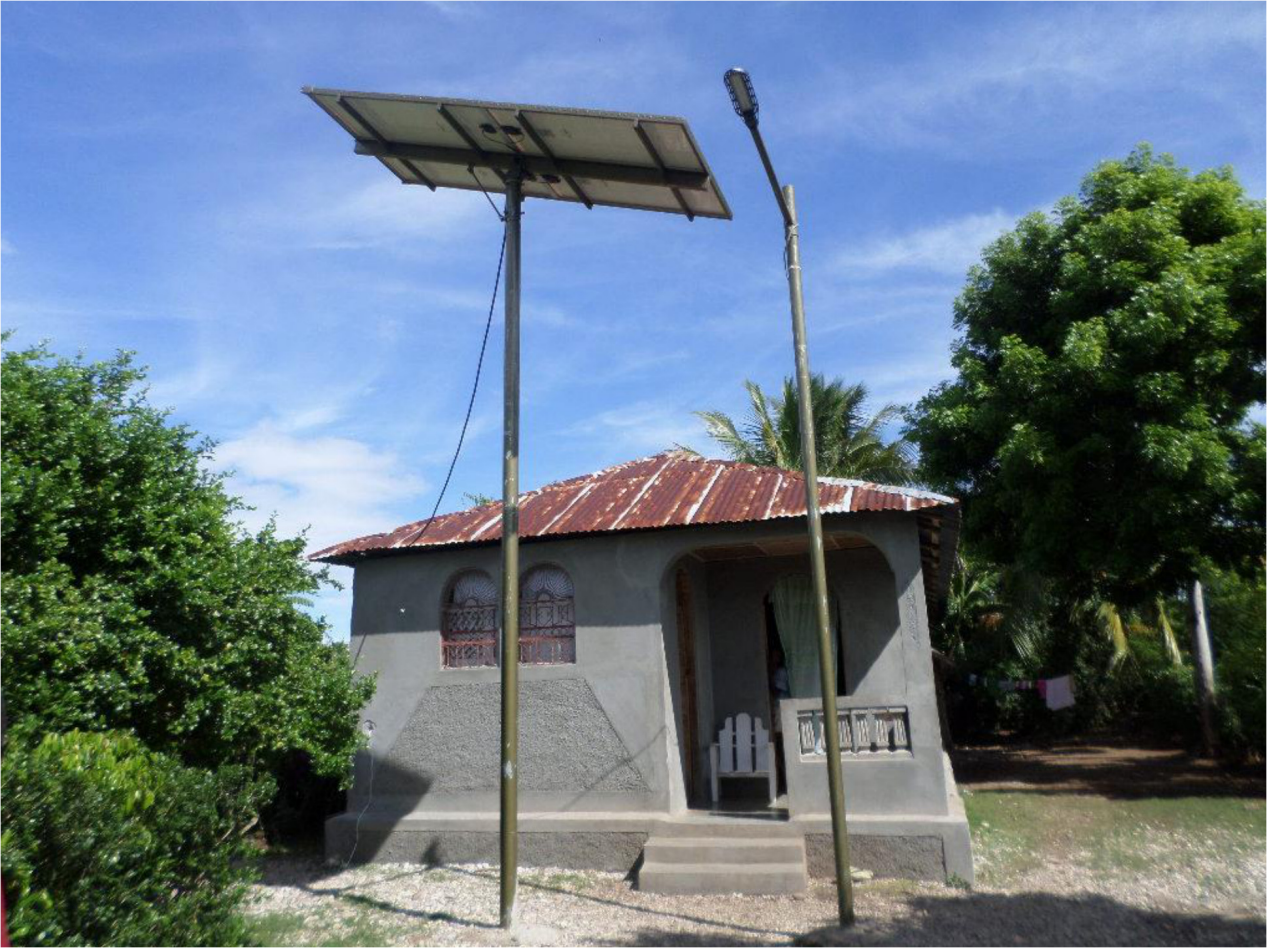
Solar panels at an outpost in Haiti. Electricity is fed to refrigerators inside the post to ensure cold chain for canine rabies vaccine.

Although canine mass vaccination was planned for 2013, no vaccination campaign was conducted. Haitian authorities relied on funds donated by the World Bank for the purchase of approximately 500,000 doses of inactivated, injectable vaccine (IMRAB by Merial). Although the funds were awarded in April 2013, the vaccine only arrived in 2014. At the time of writing (November 2014), dog vaccination was finally underway. It started in September 2014 and is expected to terminate in January 2015. This four month period for the completion of the campaign responds purely to the lack of enough manpower to deliver a faster implementation. To support the vaccination campaign, MARNDR is implementing a solar-powered cold chain (Fig 1), over and above the available cold chain facilities in MSPP field premises, previously used to support dog vaccination campaigns.

### Post Exposure Prophylaxis

At the individual patient level, post exposure prophylaxis (PEP) consists of i) local treatment of the wound, ii) vaccination, and iii) administration of immunoglobulin (if indicated) [3]. At the programme level, several components are critical to this capacity: i) adequate and prompt recognition of the need of PEP by the public, if exposed, and by health officials, ii) prompt and sufficient availability of PEP of quality, and iii) adequate follow-up of PEP use. Awareness by health officials of the need of PEP after a dog bite can only be achieved via timely and spatially relevant communication efforts about the epidemiological situation of rabies. On this note, dog bites are now, together with six notifiable conditions, part of the daily reporting requirements from 114 health units (out of more than 900 in the country) to a central epidemiology unit in Port-au-Prince, the capital. This intensive reporting pressure on health officials should increase rabies awareness across the health system, and lead to an increase in the number of dog attacks reported. The aggregation of this information, analysis, and feedback to the sources on a regular basis would further improve awareness among health officials of the rabies risks.

As reported by health officials, 1,657 dog bites were recorded in 2013 by the 114 sentinel health centers. For a population of approximately 10 million people, this results in a 0.016% of the population bitten by dogs; five times lower than the 0.08% average reported by the countries that attended the 14^th^ meeting of National Rabies Programme Managers (REDIPRA in Spanish) in Lima (Perú) in 2013 [7], and many more times lower than the 1.8% reported in other countries where canine rabies is also endemic [8]. Analyses of the number of animal aggressions reported by the public may inform the level of awareness within the population with regard to the risk of rabies. A simple and short questionnaire about the motivation of the visit by the dog bitten patient, i.e., whether it was related to fear of rabies, could be administered to refine this inference. Further checks on the quality of the data should help explain apparent reporting artifacts, such as the fact that all registered victims of dog attacks were five years old or older, a finding that challenges results elsewhere that consistently report young children as the main victims of dog bites [9,10,11]. The surveillance artifact may hide greater under-reporting of rabies cases if this population group is not captured by the bite registration mechanisms, and should trigger questions as to where do children younger than five years old go for bite treatment.

At the time of the review, the MSPP had more than 15,000 doses of human vaccine for PEP in stock. These vaccines belonged to a lot of 20,000 Vero cell rabies vaccines for intramuscular administration donated by Brazil in 2013. This volume of vaccine should be enough to cover the needs of the country for at least two years given the current use rate of 8,000 doses per year. In addition to sufficient numbers, vaccine must be stored in adequate conditions and in such a way that allows its prompt availability across the country. A national consultant hired by PAHO/Haiti visited twelve sites in Port-au-Prince during the last months of 2013 and reported adequate cold chain. We had no data about the conditions outside the capital.

At an annual use rate of 8,000 human vaccine doses (as per PAHO/Haiti’s warehouse records), the country could face a surplus of several thousand doses by the end of their shelf life in early 2015. Solutions to the adequate use of this potential excess of vaccine, before they reach their expiration date, might contemplate increasing the distribution of vaccine to areas where it is not currently available, e.g., outside the capital. According to MSPP officials, all large hospitals in the country (between 40 and 50) had stocks of human vaccine. Ensuring that vaccine regimens after exposure are taken fully by as many patients as possible is also critical. MSPP officials confirmed that they do not record this key performance indicator. At the time of the mission, the amount and availability of any stock of immunoglobulin was not ascertained.

### Pre-Exposure Prophylaxis

The 2013 WHO expert consultation on rabies report [3] indicates the administration of pre-exposure prophylaxis for those populations at continuous or high risk of rabies, especially children. Although the current stock of vaccine could support such strategy, and indirectly reduce the potential surplus of vaccine by 2015, the logistics of the distribution, rationale for application, and the required follow-up would make this operation impossible given the current available resources.

### Outbreak Control Activities

This capacity comprises surveillance, awareness raising, and control specific activities, within a defined time and space context, implemented following the confirmation of a case of rabies. These can include enhanced surveillance of animal populations at risk, ring vaccination, and awareness campaigns for the population. The rabies national plan provides protocols to guarantee the exchange of information between MSPP and the MARNDR after the occurrence of rabies events. For example, the communication of dog attacks by the MSPP to the MARNDR, for the latter to initiate surveillance and control activities, occurs after a number of dog attacks are recorded for a specific area. At the time of this review, it was not possible to ascertain the criteria, e.g., number of dogs and period of time that trigger this communication, nor was it possible to verify the completeness of epidemiological reports around the cases.

Reactive vaccination of susceptible animals after confirmation of rabies within the local community is also part of these case control activities, however, the amount of canine vaccine available for this purpose at the time of the mission, around 1,000 doses, appeared insufficient.

### Education

Rabies educational capacity comprises activities towards health professionals and the general public to enhance and maintain awareness of rabies risks. To this end, the MSPP conducted a training activity for 40 health officials of the central region on PEP in 2013. This training followed four similar courses delivered in 2012. It was not possible to ascertain if these activities were all part of a training plan.

MSPP and PAHO/Haiti officials stated the critical importance of training for health officials on PEP, data form completion, etc., and stressed the ongoing need for these activities given the high staff turnover. Training on PEP application should help to change the current practice of delaying prophylaxis after exposure if the animal can be observed. This practice can lead to fatal delays [3] and it needs to be replaced with the immediate application of PEP after exposure even if the animal can be observed.

For MARNDR officials, there are currently no training plans, although the CDC trained more than 30 veterinary officials in June 2013, and delivers regular training to personnel of the MARNDR laboratory responsible for rabies diagnosis.

### Identification of Other Risks

Cases of canine rabies together with the significant numbers of mongoose cases reported from the Dominican Republic could be seen as a threat to Haiti’s rabies status [7]. Despite this being a genuine concern, and an indication of a source of rabies in Haiti’s wildlife, it is not high in the list of priorities given the many limitations affecting the other capacities. Any medium- or long-term strategy in the future must contemplate the wildlife source. In the short term, enhanced communication and coordination of control efforts with the neighbouring Dominican Republic would bring benefits to the two countries.

## Discussion

Despite having a National Rabies Plan for the period 2007–2012, and an evaluation of it in 2011, little of the plan could be delivered due to insufficient resources at the MSPP and MARNDR. Whereas in other countries in the region the main interest resides on how rabies control activities are operationalised, as the focus is on the optimization of well-established processes, in Haiti this is not the case, as some basic rabies capacities are still wanting. This is true for the episodic mass canine vaccination campaigns, and the lack of diagnostic facilities for human rabies cases. Given that canine vaccination is the most effective measure to control dog-mediated human rabies, the intermittent nature of the mass campaigns and their insufficient coverage will not allow for the control of the disease any time soon, let alone its elimination.

The inconsistent and insufficient canine vaccination campaigns will also stop short of delivering a number of related positive effects associated with vaccination, e.g., increased awareness in the population due to the high visibility of the campaigns, and a sense of purpose among the officials responsible for rabies control. Not only that, the ongoing occurrence of dog cases due to low levels of immunity in the population, despite large logistic efforts, can easily lead to frustration among officials and hamper participation by the population [12].

Following recommendations from the 14^th^ REDIPRA meeting [7] canine rabies vaccine has been recently added to PAHO’s Revolving Fund (RF). Countries in the region can now request canine vaccine from the RF. Despite the advantages provided by the inclusion of the canine vaccine in the RF, e.g., consistent vaccine quality and more competitive prices, the evidence to support the allocation of funds within the MSPP and MARNDR to purchase rabies vaccine is not sufficient. This evidence can only be gathered by means of adequate surveillance to inform the burden of rabies against that of other diseases.

Although there are models that estimate a much larger number of human rabies cases in Haiti [3], these cases remain unobserved, and lead to no reaction by the already overstretched and under-resourced Haitian health system. This appears logical as the impact of these estimated, but unobserved, cases is difficult to demonstrate relative to that of other conditions that provide counts of actual data and lead to health care expenditure. Disease estimates adjusted for under-ascertainment trigger little policy action [13], at least at the operational level, and are not a substitute for actual surveillance data. The dog surveillance work conducted by the MARNDR with technical support by the CDC is, therefore, critical. Short of reliable records on the real count of human rabies cases, measures of rabies risk approximated by the dog surveillance figures, in combination with dog bite data, can provide an indication of the much higher level of rabies exposure in the population.

Surveillance findings need to be disseminated to attain their objective: inform and influence decision, at as many levels as possible. There is value in reporting back to the sources of data for awareness and motivation reasons, to local authorities to inform strategic and operational decisions pertaining to the rabies programme, and to international forums to raise awareness about the situation in Haiti. To this end, Haiti should return to the regular reporting of surveillance figures and details of the cases to the regional database on rabies (SIRVERA) held at PANAFTOSA [6]. By having up-to-date data by country and aggregated figures, PAHO can report progress towards the goal of elimination in the region, and identify countries in need of greater focus, such as Haiti. At this critical stage in the race to elimination, with fewer cases and diminishing resources and available skill pool in the region, collecting and sharing as much information as possible on the epidemiology and, critically, on the processes leading to the surveillance and control of the disease remains as important as ever.

Following from the recommendations of the last REDIPRA meeting [7], PAHO coordinated in 2014 a proficiency exercise for 35 laboratories from 23 countries to assess regional consistency in direct fluorescent antibody techniques. Haiti’s MARNDR reference laboratory has participated in this exercise. This shows the commitment of the Haitian authorities to improve their processes. This willingness should be extended to seek the utilization of the MARNDR laboratory premises for the diagnoses of human rabies cases, over and above legitimate administrative and organizational concerns. On this note, as of July 2014, there is discussion about developing a plan for a joint human and animal rabies diagnostic facility in the North Department. If successful, this will represent the second animal rabies diagnostic facility and first human rabies diagnostic facility in the country. The initiative to create a joint human/animal rabies diagnostic laboratory would maximize the use of the limited human resource and infrastructure capital. Given the current low recognition of human rabies, and the fact that the majority of routine diagnostic testing will be on animal samples, it is not feasible to create a diagnostic facility solely devoted to human diagnostics, nor train personnel in human rabies diagnostics alone.

The Brazilian Ministry of Health is willing to consider far-reaching and more effective ways of collaborating with Haiti, extending the ad-hoc donations of vaccine to on-the-ground technical support on areas such as vaccine campaigns planning and delivery, PEP trainings to health officials, and others. Brazil’s technical contribution will only add to the increasing need to provide a strategic framework, with clear objectives, i.e., the control of the disease in the canine population first to ensure the efficient elimination of dog mediated human cases, and coordination between donors.

The impact of international technical missions like the one reported here appears reduced, at least in terms of observing rapid changes in response to limitations that are well known by the local officials. The benefit could come in the form of notes like this and their dissemination: to increase awareness among international donors, senior authorities in Haiti, and within PAHO. A planned communication campaign is just one of the activities contained in the strategic document, being drafted at the time of writing, to support a rabies task force in Haiti. Key to this strategy, and responding to a critical lack of this capacity, is the need for coordination among the multiple partners that deliver rabies activities in Haiti. Better integration among partners should lead to greater impact and more efficient use of resources. Coordination and speaking with one voice before Haitian authorities would also increase the critical mass of rabies control efforts and deliver a more consistent and rotund message. The funding of a rabies coordinator/rabies champion, who will work primarily towards ensuring regular and sufficient canine vaccination campaigns and prompt availability of human vaccine for the next two to three years, is being discussed at the time of writing.

The current situation in Haiti clearly impacts on the region’s 2015 elimination goal [1]. This will not be achieved. The countries of the region will have to discuss and agree on a new goal that recognizes the distinct situations across the region, and strategies to that effect. Most of the countries and areas, where dog-mediated human rabies is no longer a public health problem, will benefit from strategies oriented to efficiency gains and enhanced knowledge management to ensure institutional memory and experience dissemination. These efforts should also benefit the reduced number of countries and areas, such as Haiti, where rabies remains a public health concern. Specific plans for dog rabies control, such as those described above for Haiti, will require contributions from a number of regional partners and neighbouring countries. The forthcoming 2015 REDIPRA meeting will provide the platform to hold these discussions and agree on a new regional goal.

A general limitation of the findings described here is that they originate from unstructured questions, observations, and notes from meetings and field visits. A more comprehensive platform to collect precise information on the levels of performance within the capacities, as well as on process-related indicators, is the subject of current work by PANAFTOSA.

**Box 1. Key Learning Points**

- Dog rabies is present in Haiti and, as a result, the risk to the human population remains high.
- Control of dog-mediated human rabies in Haiti is not yet in sight due to inadequate resources to sustain sufficient control efforts. Rabies surveillance is wanting and the real impact of the disease is unknown.
- These overwhelming limitations reduce the impact of Haitian officials’ efforts towards the control of the disease.
- The contribution of international partners in terms of financial resources and technical assistance is not enough to sustain activities on the ground to control the disease. In addition to that, the coordination of their efforts remains an area for improvement.
- Unless the international community ramps up their efforts to support rabies control in Haiti, the goal of dog-transmitted human rabies elimination in the American region appears compromised by the situation in Haiti.

**Box 2. Top Five Papers**

- Meslin F.X., Briggs D.J. Eliminating canine rabies, the principal source of human infection: what will it take? Antiviral Research 2013, 98 (2), 291–296.
- Vigilato M.A., Clavijo A., Knobl T., Tamayo H.M., Cosivi O., Schneider M.C., Leanes L.F., Belotto A.J., Espinal M.A. Progress towards eliminating canine rabies: policies and perspectives from latin American and the Caribbean. Phil Trans R Soc B 2013, 368: 20120143.
- Clavijo A., Del Rio Vilas V.J., Mayen F.L., Yadon Z.E., Beloto A.J., Vigilato M.A., Schneider M.C., Cosivi O. Gains and future road map for the elimination of dog transmitted rabies in the Americas. Am. J. Trop. Med. Hyg. 2013; 89(6), 1040–2.
- Fooks A.R., Banyard A.C., Horton D.L., Johnson N., McElhinney L.M., Jackson A.C. Current status of rabies and prospects for elimination. The Lancet 2014. http://dx.doi.org/10.1016/S0140-6736(13)62707-5
- Cleaveland S., Lankester F., Townsend S., Lembo T., Hampson K. Rabies control and elimination: a test case for One Health. Vet Rec 2014, 175: 188–193.
